# Supplementary material for: Environmental, Social, and Health Burdens in relation to Sleep-Disordered Breathing among Patients of Community-Based Health Centers in the United States
Source: medRxiv. 2025 Oct 2:2025.10.01.25337104. Preprint. [Version 1] doi: 10.1101/2025.10.01.25337104 (PMC12622111; doi:10.1101/2025.10.01.25337104)
Supplement: Supplement 1 [file NIHPP2025.10.01.25337104v1-supplement-1.pdf]

Supplemental Materials

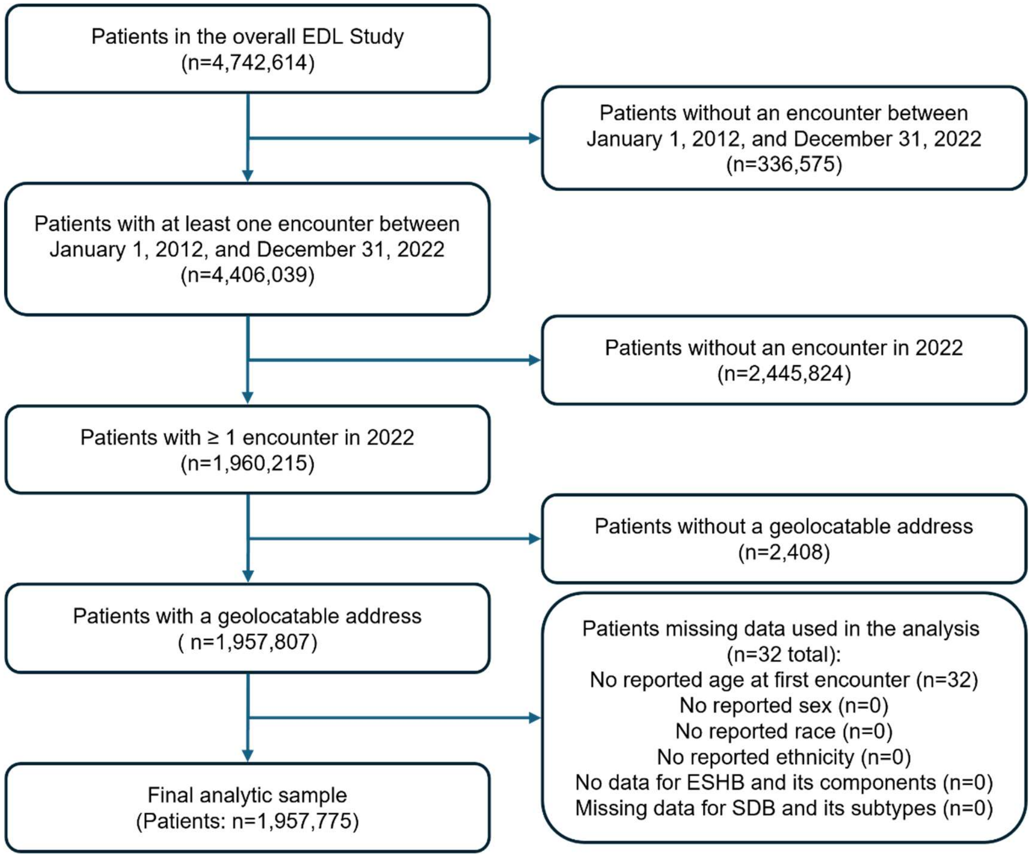

eFigure 1. A flowchart of the study inclusions/exclusion criteria of the association between Environmental, Social, and Health Burden (ESHB) and sleep-disordered breathing (SDB)

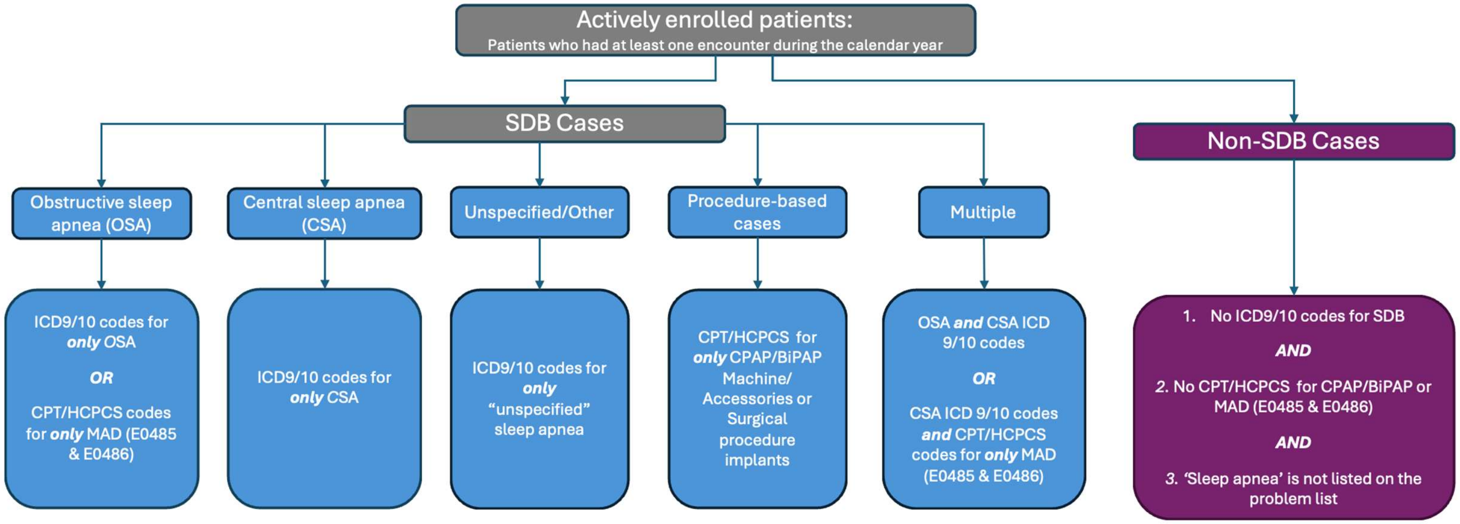

Diagnosis codes		
**Diagnosis**	**ICD-9**	**ICD-10**
Central sleep apnea	327.27, 327.21	G47.37, G47.31
Obstructive sleep apnea	327.23	G47.33
Other/unspecified sleep apnea	327.2, 327.20, 780.57, 780.53, 780.51, 327.29, 786.03	G47.30, G47.39, R06.81, G47.3

Table 2. Surgical/procedural codes (CPT/HCPCS)	
**Machine**	E0601 E0485 E0486 94660 E0470· E0471· E0472 E0561 E0562
**Accessories**	A7030 A7031 A7034 A7044 A7027 A7028 A7029 A7032 A7033 A7035 A7036 A7037 A4604 A7038 A7039 A7045 A7046 E1399
**Mandibular Advancement Device**	E0485, E0486
**Surgical Procedures Implants**	64568 0466T 61886 61888 64569 64570 64585 0467T 0468T

Abbreviations:
CSA = Central sleep apnea
MAD = Mandibular Advancement Device
OSA = Obstructive sleep apnea

eFigure 2. Flowchart of sample selection

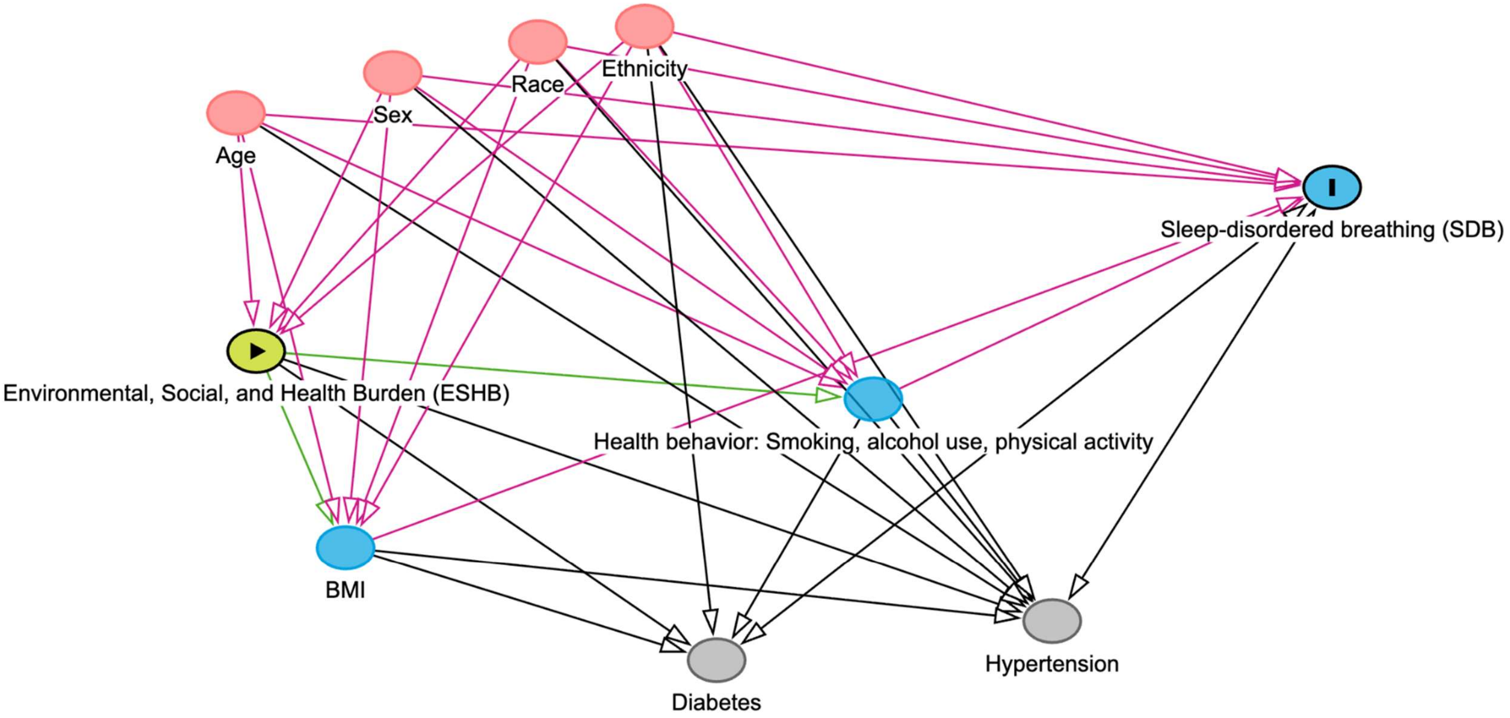

eFigure 3. Directed Acyclic Graph (DAG) for the association between Environmental, Social, and Health Burden (ESHB) and sleep-disordered breathing (SDB)

eTable 1. Comparison of socioeconomic characteristics between the included and excluded samples in the study (n=1,957,775)

	Included	Excluded	Overall	P for group comparison
**N**	1,787,524 (91.3)	170,251 (8.7)	1,957,775 (100.0)	
**Age category**				
18-34 years	605,179 (33.9)	52,174 (30.6)	657,353 (33.6)	<0.01
35-49 years	490,767 (27.5)	43,845 (25.8)	534,612 (27.3)	<0.01
≥50 years	691,578 (38.7)	74,232 (43.6)	765,810 (39.1)	<0.01
**Sex**				
Men	719,307 (40.2)	74,953 (44.0)	794,260 (40.6)	<0.01
Women	1,066,752 (59.7)	95,105 (55.9)	1,161,857 (59.3)	<0.01
Unknown/Missing	1,465 (0.1)	193 (0.1)	1,658 (0.1)	<0.01
**Race**				
AI/AN	21,061 (1.2)	2,465 (1.4)	23,526 (1.2)	<0.01
Asian	115,244 (6.4)	6,429 (3.8)	121,673 (6.2)	<0.01
BAA	309,729 (17.3)	25,487 (15.0)	335,216 (17.1)	<0.01
NHPI	7,524 (0.4)	507 (0.3)	8,031 (0.4)	<0.01
White	1,031,663 (57.7)	105,168 (61.8)	1,136,831 (58.1)	<0.01
Multiple	18,183 (1.0)	1,547 (0.9)	19,730 (1.0)	<0.01
Unknown/Missing	284,120 (15.9)	28,648 (16.8)	312,768 (16.0)	<0.01
**Ethnicity**				
Hispanic	606,242 (33.9)	47,840 (28.1)	654,082 (33.4)	<0.01
Non-Hispanic	1,024,478 (57.3)	101,927 (59.9)	1,126,405 (57.5)	<0.01
Unknown/Missing	156,804 (8.8)	20,484 (12.0)	177,288 (9.1)	<0.01
**Veteran**				
Yes	828,386 (46.3)	50,160 (29.5)	878,546 (44.9)	<0.01

	Included	Excluded	Overall	P for group comparison
No	26,166 (1.5)	3,694 (2.2)	29,860 (1.5)	<0.01
Missing	932,972 (52.2)	116,397 (68.4)	1,049,369 (53.6)	<0.01
**Unhoused**				
Yes	1,486,859 (83.2)	133,891 (78.6)	1,620,750 (82.8)	<0.01
No	60,023 (3.4)	9,509 (5.6)	69,532 (3.6)	<0.01
Missing	240,642 (13.5)	26,851 (15.8)	267,493 (13.7)	<0.01
**Health insurance**				
Private	318,069 (17.8)	30,802 (18.1)	348,871 (17.8)	<0.01
Medicaid	679,913 (38.0)	50,031 (29.4)	729,944 (37.3)	<0.01
Medicare	196,316 (11.0)	22,690 (13.3)	219,006 (11.2)	<0.01
Military	285 (0.0)	38 (0.0)	323 (0.0)	0.05
Other public	10,631 (0.6)	482 (0.3)	11,113 (0.6)	<0.01
Multiple	69,617 (3.9)	4,135 (2.4)	73,752 (3.8)	<0.01
Uninsured	205,256 (11.5)	24,471 (14.4)	229,727 (11.7)	<0.01
Unknown/Missing	307,437 (17.2)	37,602 (22.1)	345,039 (17.6)	<0.01
**U.S. Region**				
Northeast	256,111 (14.3)	<11	256,111 (13.1)	<0.01
South	201,956 (11.3)	<11	201,956 (10.3)	<0.01
Midwest	279,836 (15.7)	<11	279,836 (14.3)	<0.01
West	1,049,621 (58.7)	<11	1,049,621 (53.6)	<0.01
Unknown/Missing	<11	170,251 (100.0)	170,251 (8.7)	<0.01
**Federal poverty level percentage**				
<25%	726,178 (40.6)	64,763 (38.0)	790,941 (40.4)	<0.01
25-49%	70,707 (4.0)	4,991 (2.9)	75,698 (3.9)	<0.01

	Included	Excluded	Overall	P for group comparison
50-99%	268,173 (15.0)	20,580 (12.1)	288,753 (14.7)	<0.01
100-137%	142,187 (8.0)	11,887 (7.0)	154,074 (7.9)	<0.01
>137%	259,951 (14.5)	25,410 (14.9)	285,361 (14.6)	<0.01
Missing	320,328 (17.9)	42,620 (25.0)	362,948 (18.5)	<0.01
**Smoking**				
Current smoker	248,504 (13.9)	22,459 (13.2)	270,963 (13.8)	<0.01
**Physical activity**				
<10 minutes	34,668 (1.9)	2,357 (1.4)	37,025 (1.9)	<0.01
10-150 minutes	25,507 (1.4)	1,778 (1.0)	27,285 (1.4)	<0.01
>150 minutes	25,460 (1.4)	1,881 (1.1)	27,341 (1.4)	<0.01
Missing	1,701,889 (95.2)	164,235 (96.5)	1,866,124 (95.3)	<0.01
**Alcohol/Substance abuse**				
No	1,522,445 (85.2)	150,299 (88.3)	1,672,744 (85.4)	<0.01
Yes	265,079 (14.8)	19,952 (11.7)	285,031 (14.6)	<0.01
Former smoker	207,596 (11.6)	17,810 (10.5)	225,406 (11.5)	<0.01
Never smoker	913,007 (51.1)	77,936 (45.8)	990,943 (50.6)	<0.01
Unknown/Missing	418,417 (23.4)	52,046 (30.6)	470,463 (24.0)	<0.01
**Body Mass Index**				
Recommended (<25 kg/m^2)	319,970 (17.9)	30,485 (17.9)	350,455 (17.9)	0.95
Overweight (25-30 kg/m^2)	405,933 (22.7)	36,796 (21.6)	442,729 (22.6)	<0.01
Obesity (>30 kg/m^2)	563,968 (31.6)	48,460 (28.5)	612,428 (31.3)	<0.01
Unknown/Missing	497,653 (27.8)	54,510 (32.0)	552,163 (28.2)	<0.01
**Hypertension**				
No	657,823 (36.8)	54,752 (32.2)	712,575 (36.4)	<0.01

	Included	Excluded	Overall	P for group comparison
Yes	940,972 (52.6)	91,637 (53.8)	1,032,609 (52.7)	
Missing	188,729 (10.6)	23,862 (14.0)	212,591 (10.9)	<0.01
**Diabetes**				
No	1,462,495 (81.8)	144,213 (84.7)	1,606,708 (82.1)	<0.01
Yes	325,029 (18.2)	26,038 (15.3)	351,067 (17.9)	<0.01
**Environmental, Social, and Health Burden**	0.591 [0.333, 0.802]	NA	0.591 [0.333, 0.802]	NA
Missing	<11	170,251 (100.0)	170,251 (8.7)	<0.01
**Environmental Burden Module**	0.515 [0.252, 0.751]	NA	0.515 [0.252, 0.751]	NA
Missing	<11	170,251 (100.0)	170,251 (8.7)	<0.01
**Social Vulnerability Module**	0.684 [0.429, 0.864]	NA	0.684 [0.429, 0.864]	NA
Missing	<11	170,251 (100.0)	170,251 (8.7)	<0.01
**Health Vulnerability Module**	0.200 [0, 0.600]	NA	0.200 [0, 0.600]	NA
Missing	<11	170,251 (100.0)	170,251 (8.7)	<0.01
**Any Sleep Disordered Breathing**				
No	1,688,928 (94.5)	161,990 (95.1)	1,850,918 (94.5)	<0.01
Yes	98,596 (5.5)	8,261 (4.9)	106,857 (5.5)	<0.01
**Central Sleep Apnea**				
No	1,787,011 (100.0)	170,202 (100.0)	1,957,213 (100.0)	0.99
Yes	513 (0.0)	49 (0.0)	562 (0.0)	0.99
**Obstructive Sleep Apnea**				
No	1,719,443 (96.2)	164,413 (96.6)	1,883,856 (96.2)	<0.01
Yes	68,081 (3.8)	5,838 (3.4)	73,919 (3.8)	<0.01
**Other/unspecified Sleep Apnea**				
No	1,762,028 (98.6)	168,116 (98.7)	1,930,144 (98.6)	<0.01

	Included	Excluded	Overall	P for group comparison
Yes	25,496 (1.4)	2,135 (1.3)	27,631 (1.4)	<0.01
**Multiple sleep apneas**				
No	1,786,939 (100.0)	170,194 (100.0)	1,957,133 (100.0)	0.87
Yes	585 (0.0)	57 (0.0)	642 (0.0)	0.87
**Procedure-based cases**				
No	1,783,603 (99.8)	170,069 (99.9)	1,953,672 (99.8)	<0.01
Yes	3,921 (0.2)	182 (0.1)	4,103 (0.2)	<0.01

Abbreviations: AI/AN=American Indian/Alaskan Native, BAA= Black/African American, NHPI=Native Hawaiian/Pacific Islander.

Data are presented as n (%).

Supplemental Text

METHODS

Data sources

Patient data were obtained from an electronic health record (EHR) data from the Across a National Community Health Center Network (ADVANCE), led by OCHIN (https://www.pcori.org/research-results/2015/accelerating-data-value-across-national-community-health-center-network-advance). [1] ADVANCE includes more than 144 independent health systems and over five million patients seen at safety-net and Federally Qualified Health Center (FQHC) clinics, making it a nationally representative Clinical Data Research Network (CDRN). [1] The OCHIN is a national nonprofit health technology organization that includes more than 34,500 providers at over 2,000 community-based health centers across 40 U.S. states. Among patients included in OCHIN, 55% were at or below the federal poverty level, 80% were uninsured or relied on public health insurance, and 16.5% resided in rural communities. [2] These patients are publicly insured or underinsured and have historically been underrepresented in research due to limited access to medical resources stemming from poverty and a general distrust of health care systems. [1] The population's relatively low income and racial and ethnic diversity provide a meaningful representation of individuals experiencing health disparities. [3,4]

Exposure assessment: Environmental, Social, and Health Burden (ESHB)

The U.S. Department of Health and Human Services' Office of Environmental Justice and the Centers for Disease Control and Prevention (CDC) and Agency for Toxic Substances and Disease Registry (ATSDR) developed a metric at the census tract level that integrates environmental, social, and health indicators. [5] These indicators are from various sources, including multiple federal agencies, such as the U.S. Census Bureau, the U.S. Environmental Protection Agency, the U.S. Mine Safety and Health Administration, and the CDC. The ESHB ranks census tracts on 10 different domains (racial/ethnic minority status, socioeconomic status, household characteristics, housing type, air pollution, potentially hazardous and toxic sites, built environment, transportation infrastructure, water pollution, pre-existing chronic disease burden) categorized under three main burdens/vulnerabilities, which represent environmental burden (i.e., 17 indicators reflecting air pollution, potentially hazardous and toxic sites, built environment, transportation infrastructure, and water pollution), social vulnerability (i.e., 14 indicators reflecting racial/ethnic minority status,

socioeconomic status, household characteristics, and housing type), and health vulnerability (i.e., five indicators reflecting pre-existing chronic disease burden). [5] Accordingly, there are three submodules: the Environmental Burden Module [EBM], the Social Vulnerability Module [SVM], and the Health Vulnerability Module [HVM]. An overall ESHB score was computed by summing the ranked scores of three modules. The sum of the three module rankings (range: 0-3) was converted to a percentile rank (range: 0-1) across all U.S. census tracts, where higher values indicate higher vulnerability.

Potential Confounders

Sociodemographic characteristics of patients, including age category in 2022 (categorized as 18-34, 35-49, 50 years and older), sex (man, woman), race (American Indian/Alaskan Native [AI/AN], Asian, Black/African American [BAA], Native Hawaiian/Pacific Islander [NHPI], White, multiple, and unknown), and ethnicity (Hispanic, Non-Hispanic, unknown). Health behavior and clinical characteristics including body mass index (BMI; diagnosed at the closest visit prior to or during 2022), smoking status (the closest non-missing data to first encounter in 2022), alcohol and substance use status (ever reported or diagnosed prior to or during 2022), physical activity (measured at the closest visit prior to or during 2022), and comorbidities were obtained. Obesity (BMI ≥30 kg/m^2) was identified using ICD-9/10-CM codes (E66.9). Smoking status was a self-reported variable using data from the general information of patients. Alcohol and substance use disorders were identified using ICD-9/10 codes, a Drug Abuse Screening Test (DAST-10) score ≥3, or an Alcohol Use Disorders Identification Test - Consumption (AUDIT-C) score ≥3 for women or ≥4 for men. [6,7] Patients meeting any of these criteria were classified as positive (“yes”) for alcohol or substance abuse, while alcohol or substance abuse was defined as “no” among patients with no indicated ICD codes and who met criteria as ‘negative’ for all tests (DAST-10 <3, AUDIT-C <3 for women or <4 for men). Patient-reported physical activity status was categorized into three groups based on the Physical Activity Guidelines for adults and older adults, which recommend at least 150 minutes to 300 minutes a week of moderate-intensity, or 75 minutes to 150 minutes a week of vigorous-intensity aerobic physical activity, or an equivalent combination of moderate- and vigorous-intensity aerobic activity. [8] The cut points were used because intensity of physical activity was not captured in the EHR. Thus, the three categories were: physically inactive (<10 minutes), insufficiently active (10-150 minutes/week), and physically active (weekly physical activity >150 minutes/week). Comorbidities included hypertension (at first encounter, including prior to 2022) and diabetes (ever diagnosed prior to or during 2022). Hypertension was defined as ≥1 Systolic BP reading ≥130 mmHg, diastolic BP reading ≥80 mmHg, hypertension medication, or ICD9/10 diagnosis. Types 1 and 2 diabetes

were defined as ever diagnosed with diabetes (by the time of the 2022 assessment) and identified using the Centers for Medicare and Medicaid Services Chronic Conditions Data Warehouse (CMS CCDW) codes. [9]

Sensitivity analysis

As a sensitivity analysis, we examined Bonferroni-corrected p-values and confidence intervals to adjust for multiple comparisons. [10] Bonferroni multiple testing corrections for the stratified models are reported in the supplemental materials. We further excluded patients with a history of alcohol or substance use, as studies suggest a positive association between substance abuse and sleep disorders. [11,12] This analysis helped ensure the robustness of our findings by confirming whether excluding these individuals would alter the main results.

RESULTS

Patient characteristics

For ESHB submodules, EBM ranks were lower in patients with SDB (including all SDB subtypes); however, SVM was highest among procedure-based cases, and HVM was consistently higher among patients with SDB (including its subtypes).

Environmental, social, and health burden and prevalence of SDB

Each 0.1-unit increase in EBM percentile rank was associated with a lower prevalence of any SDB (PR=0.97 [0.97–0.97]), OSA (PR=0.97 [0.97–0.97]), and OUSA (PR=0.99 [0.99–0.99]), multiple sleep apneas (PR=0.89 [0.86–0.91]), and procedure-based cases (PR=0.94 [0.93–0.95]). Each 0.1-unit increase in SVM percentile rank was associated with a higher prevalence of any SDB (PR=1.02 [1.01–1.02]), OSA (PR=1.02 [1.01–1.02]), multiple sleep apneas (PR=1.06 [1.02–1.09]), and procedure-based cases (PR=1.08 [1.07–1.10]). Each 0.1-unit increase in HVM percentile rank was associated with a higher documented prevalence of any SDB (PR=1.02 [1.02–1.02]), OSA (PR=1.02 [1.02–1.02]), OUSA (PR=1.02 [1.02–1.03]), multiple sleep apneas (PR=1.04 [1.01–1.06]), and procedure-based cases (PR=1.04 [1.03–1.05]). There were no associations with CSA.

Effect modification of associations between EBM, SVM, and HVM in relation to SDB

By age

EBM was more strongly associated with a lower prevalence of any SDB and OSA among the oldest age group ($PR_{18\text{-}34\ years}$=0.98 [0.97–0.99], $PR_{35\text{-}49\ years}$=0.97 [0.97–0.98], $PR_{\geq 50\ years}$=0.97 [0.97–0.97], $P_{interaction}$<0.01; eTable 5). SVM and HVM were more strongly associated with any SDB and specific subtypes (i.e., OSA and OUSA) among the youngest age group (e.g., SVM–SDB: $PR_{18\text{-}34\ years}$=1.03 [1.02–1.04], $PR_{35\text{-}49\ years}$=1.02 [1.02–1.03], $PR_{\geq 50\ years}$=1.01 [1.01–1.02]; HVM-SDB: $PR_{18\text{-}34\ years}$=1.04 [1.03–1.04], $PR_{35\text{-}49\ years}$=1.03 [1.03–1.04], $PR_{\geq 50\ years}$=1.02 [1.01–1.02]; eTable 6-7).

By sex

There were stronger protective associations between EBM and SDB, OSA, and OUSA among men (e.g., SDB: PR_{men}=0.96 [0.96–0.97], PR_{women}=0.98 [0.98–0.98]; eTable 5). Consistent with ESHB, SVM and HVM were more strongly related to SDB, OSA, and OUSA in women than men (eTable 6-7).

By race

EBM had stronger protective associations with SDB and OSA among AI/AN individuals (SDB: $PR_{AI/AN}$=0.94 [0.93–0.96] and OSA: $PR_{AI/AN}$=0.93 [0.91–0.95]) and with multiple sleep apneas among multiracial individuals ($PR_{Multiracial}$=0.72 [0.55–0.94]) than White patients (SDB: PR_{White}=0.97 [0.97–0.97], OSA: PR_{White}=0.96 [0.96–0.97], Multiple: PR_{White}=0.94 [0.92–0.96]). Among BAA, EBM was also associated with a lower prevalence of procedure-based cases (PR=0.86 [0.83–0.88]; eTable 5). Overall, the patterns of association for SVM and HVM with SDB or OSA were consistent with those observed for the ESHB-SDB association (eTable 6-7), with one subtle difference: for the SVM and SDB relationship, PRs were comparable between AI/AN and White participants but lower among Asian participants (SDB: PR_{Asian}=0.99 [0.98–1.01] and OSA: PR_{Asian}=0.98 [0.96–0.99]; eTable 6). For procedure-based case prevalences, the associations with SVM and HVM were higher among Asian compared to White patients (SVM: PR_{Asian}=1.30 [1.23–1.37] and PR_{White}=1.09 [1.07–1.11]; HVM: PR_{Asian}=1.29 [1.23–1.34] and PR_{White}=1.04 [1.02–1.05]; eTable 6-7), similar to observed associations with ESHB (eTable 5).

By ethnicity

There was an inverse association between ESHB and SDB ($PR_{Hispanic}$=0.98 [0.98–0.99]), OSA ($PR_{Hispanic}$=0.99 [0.98–0.99]), and OUSA ($PR_{Hispanic}$=0.98 [0.97–0.99]) among Hispanic patients (eTable 4); EBM conferred a protective association against SDB and procedure-based cases in non-Hispanic patients, but the CIs overlapped with those observed among Hispanic patients (eTable 5). There were stronger associations between higher EBM and lower prevalence of OSA and OUSA among Hispanic patients (eTable 5). For SVM, there were inverse associations with SDB, OSA, OUSA, and procedure-based cases among Hispanic patients, whereas higher SVM was linked to higher prevalence of these outcomes among non-Hispanic patients (eTable 6). For HVM, more pronounced positive associations with SDB, OSA, OUSA, and procedure-based cases were observed in non-Hispanic individuals (eTable 7).

REFERENCES

1. DeVoe JE, Gold R, Cottrell E, et al. The ADVANCE network: accelerating data value across a national community health center network. J Am Med Inform Assoc. Jul-Aug 2014;21(4):591-5. doi:10.1136/amiajnl-2014-002744
2. OCHIN. Our OCHIN Epic patient demographics. https://ochin.org/network/patients/
3. Ayanian JZ. Community Health Centers Caring for Adults With Hypertension and Diabetes. JAMA Health Forum. Sep 3 2021;2(9):e212678. doi:10.1001/jamahealthforum.2021.2678
4. Accelerating Data Value Across a National Community Health Center Network (ADVANCE). 2024. Accessed May 5, 2024. https://www.pcori.org/research-results/2015/accelerating-data-value-across-national-community-health-center-network-advance
5. Centers for Disease Control and Prevention, Agency for Toxic Substances Disease Registry. 2022 Environmental Justice Index. Accessed July 24, https://www.atsdr.cdc.gov/placeandhealth/eji/index.html.
6. Higgins-Biddle JC, Babor TF. A review of the Alcohol Use Disorders Identification Test (AUDIT), AUDIT-C, and USAUDIT for screening in the United States: Past issues and future directions. Am J Drug Alcohol Abuse. 2018;44(6):578-586. doi:10.1080/00952990.2018.1456545

7. Shirinbayan P, Salavati M, Soleimani F, et al. The Psychometric Properties of the Drug Abuse Screening Test. Addict Health. Jan 2020;12(1):25-33. doi:10.22122/ahj.v12i1.256
8. Piercy KL, Troiano RP, Ballard RM, et al. The Physical Activity Guidelines for Americans. JAMA. Nov 20 2018;320(19):2020-2028. doi:10.1001/jama.2018.14854
9. Services CfMM. Condition categories – Chronic Conditions Data Warehouse. https://www2.ccwdata.org/web/guest/condition-categories-chronic
10. Vickerstaff V, Omar RZ, Ambler G. Methods to adjust for multiple comparisons in the analysis and sample size calculation of randomised controlled trials with multiple primary outcomes. BMC Med Res Methodol. Jun 21 2019;19(1):129. doi:10.1186/s12874-019-0754-4
11. Asadpour H, Naghibi SM, Rahimi S, et al. Prolonged Sleep Apnea in Two Patients with a History of Opium Abuse -A Case Report. Iran J Otorhinolaryngol. Mar 2020;32(109):127-131. doi:10.22038/ijorl.2020.41832.2365
12. Baldassarri SR, Chu JH, Deng A, et al. Nicotine, alcohol, and caffeine use among individuals with untreated obstructive sleep apnea. Sleep Breath. Dec 2023;27(6):2479-2490. doi:10.1007/s11325-023-02830-3

eTable 2. Detailed identification of sleep-disordered breathing (SDB) and its type

Variable	Variable specifications
SDB	Identified using (1) diagnosis codes at a study encounter, (2) procedural codes, (3) surgical codes, and (4) diagnosis codes from the medical history and conditions tables (named Problem list in the OCHIN data).<br><br>a. **Non-SDB case**: Patients without an SDB ICD 9/10 code (eTable 3) AND no CPAP/BiPAP procedure/surgical codes or Mandibular Advancement Device codes (E0485 and E0486; eTable 4) AND sleep apnea not indicated on the problem list (eTable 4).<br>b. **SDB case**: SDB ICD 9/10 code (eTable 3) OR CPAP/BiPAP procedure/surgical codes and Mandibular Advancement Device codes (E0485 and E0486eTable 3) OR sleep apnea indicated as active on the problem list (eTable 4).
SDB Type (only among SDB cases)	c. **Central sleep apnea**: central sleep apnea ICD 9/10 codes (eTable 3) AND without the presence of obstructive sleep apnea diagnosis code<br>d. **Obstructive sleep apnea**: obstructive sleep apnea ICD 9/10 codes (eTable 3) OR Mandibular Advancement Device codes (E0485 and E0486; eTable 3) AND without the presence of a central sleep apnea diagnosis code<br>e. **Procedure-based cases** (*cases identified from surgical and procedure codes*): Patients identified as an SDB case with HCPCS/CPT Codes from surgical history or procedures tables for CPAP and BiPAP Machine and Accessories or Surgical Procedure Implants (eTable 3) and NOT Mandibular Advancement Device codes (eTable 3), and no central or obstructive sleep apnea diagnosis identified with ICD 9/10 codes<br>** *Note: these patients have missing information as a construct of the EHR; this is not necessarily a distinct diagnosis group. Suggest labelling as cases identified from surgical and procedure codes, or cases missing diagnosis information, to help readers better understand the patients this group is capturing*<br>f. **Unspecified** *(suggest using "unspecified" as this group name, as "unspecified" is used for ICD codes included in this group (e.g., codes:* G47.30, G47.3, 780.57*)*: Patients with an unspecified SDB ICD 9/10 diagnosis code (eTable 3) only and no CSA or OSA ICD 9/10 code.<br>g. More than one SDB type identified (**multiple**): cases with OSA and CSA ICD 9/10 codes, or CSA ICD 9/10 and MAD HCPCS/CPT Codes. See eTable 3-4 below.

eTable 3. ICD-9/ICD-10 Codes and CPT/HCPCS for central sleep apnea (CSA), obstructive sleep apnea (OSA), and other/unspecified sleep apnea (OUSA), multiple sleep apneas, and sleep apnea diagnosed by surgical/procedural code

Diagnosis	ICD-9	ICD-10	CPT/HCPCS
Central Sleep Apnea	327.27, 327.21	G47.37, G47.31	-----
Obstructive Sleep Apnea	327.23	G47.33	E0485, E0486
Other/unspecified Sleep Apnea	327.2, 327.20, 780.57, 780.53, 780.51, 327.29, 786.03	G47.30, G47.39, R06.81, G47.3	-----
Procedure-based cases			
CPAP/BiPAP Machine	-----	-----	E0601, E0485, E0486, 94660, E0470, E0471, E0472, E0561, E0562
CPAP/BiPAP Accessories	-----	-----	A7030, A7031, A7034, A7044, A7027, A7028, A7029, A7032, A7033, A7035, A7036, A7037, A4604, A7038, A7039, A7045, A7046, E1399
Surgical Procedure Implants	-----	-----	64568, 0466T, 61886, 61888, 64569, 64570, 64585, 0467T, 0468T
Multiple	327.27, 327.21, 327.23	G47.37, G47.31, G47.33	E0485, E0486

eTable 4. Sleep apnea conditions captured in the problem list

Description	Codes
Apnea	786.03
Apnea, not elsewhere classified	R06.81
Central sleep apnea in conditions classified elsewhere	327.27, G47.37
Hypersomnia with sleep apnea, unspecified	780.53
Insomnia with sleep apnea, unspecified	780.51
Obstructive sleep apnea	327.23
Organic sleep apnea	327.2
Other organic sleep apnea	327.29
Other sleep apnea	G47.39
Primary central sleep apnea	327.21, G47.31
Sleep apnea, unspecified	G47.30, G47.3, 780.57

eTable 5. Subgroup cross-sectional associations of per 0.1-unit increase in Environmental Burden Module (EBM) ranks with sleep-disordered breathing (SDB), central sleep apnea (CSA), obstructive sleep apnea (OSA), and other/unspecified sleep apnea (OUSA), multiple sleep apneas, and procedure-based cases

	**Any SDB**		**Types of SDB**									
			**CSA**		**OSA**		**OUSA**		**Multiple sleep apneas**		**Procedure-based cases**	
	PR (95% CI)	P-value	PR (95% CI)	P-value	PR (95% CI)	P-value	PR (95% CI)	P-value	PR (95% CI)	P-value	PR (95% CI)	P-value
**Age category** [a]												
18-34 years	**0.98 (0.97, 0.99)**	<0.01	1.01 (0.92, 1.11)	0.82	**0.97 (0.97, 0.98)**	<0.01	0.99 (0.98, 1.00)	0.06	**0.80 (0.69, 0.93)**	<0.01	**0.97 (0.94, 1.00)**	0.03
35-49 years	**0.97 (0.97, 0.98)**	<0.01	0.98 (0.92, 1.05)	0.57	**0.97 (0.97, 0.98)**	<0.01	**0.99 (0.98, 1.00)**	0.01	**0.90 (0.84, 0.96)**	<0.01	**0.93 (0.90, 0.95)**	<0.01
≥50 years	**0.97 (0.97, 0.97)**	<0.01	**0.95 (0.92, 0.99)**	0.01	**0.96 (0.96, 0.97)**	<0.01	**0.99 (0.98, 1.00)**	<0.01	**0.89 (0.86, 0.92)**	<0.01	**0.94 (0.93, 0.95)**	<0.01
P for interaction	<0.01		0.47		<0.01		0.96		0.48		0.06	
**Sex** [b]												
Women	**0.98 (0.98, 0.98)**	<0.01	0.97 (0.93, 1.02)	0.19	**0.98 (0.97, 0.98)**	<0.01	1.00 (0.99, 1.01)	0.93	**0.89 (0.85, 0.93)**	<0.01	**0.94 (0.93, 0.96)**	<0.01
Men	**0.96 (0.96, 0.97)**	<0.01	0.96 (0.92, 1.00)	0.07	**0.96 (0.96, 0.96)**	<0.01	**0.98 (0.97, 0.99)**	<0.01	**0.88 (0.85, 0.92)**	<0.01	**0.94 (0.92, 0.95)**	<0.01
Unknown/Missing	0.98 (0.90, 1.08)	0.73	NE	-	0.98 (0.87, 1.10)	0.74	0.99 (0.83, 1.17)	0.87	1.43 (0.62, 3.25)	0.40	0.84 (0.49, 1.43)	0.52
P for interaction effect	<0.01		1.00		<0.01		<0.01		0.43		0.54	
**Race** [c]												

	Any SDB		Types of SDB									
			CSA		OSA		OUSA		Multiple sleep apneas		Procedure-based cases	
	PR (95% CI)	P-value	PR (95% CI)	P-value	PR (95% CI)	P-value	PR (95% CI)	P-value	PR (95% CI)	P-value	PR (95% CI)	P-value
AI/AN	**0.94 (0.93, 0.96)**	<0.01	1.04 (0.80, 1.35)	0.77	**0.93 (0.91, 0.95)**	<0.01	0.97 (0.93, 1.01)	0.12	0.97 (0.75, 1.26)	0.82	0.97 (0.89, 1.07)	0.55
Asian	**0.97 (0.96, 0.98)**	<0.01	1.02 (0.79, 1.31)	0.89	**0.97 (0.95, 0.98)**	<0.01	0.99 (0.96, 1.01)	0.26	0.88 (0.71, 1.09)	0.25	**0.90 (0.86, 0.94)**	<0.01
BAA	**0.98 (0.98, 0.99)**	<0.01	1.05 (0.95, 1.15)	0.38	**0.98 (0.98, 0.99)**	**<0.01**	1.01 (1.00, 1.02)	0.17	1.01 (0.91, 1.12)	0.86	**0.86 (0.83, 0.88)**	<0.01
NHPI	NE	-	NE	-	NE	-	NE	-	NE	-	NE	-
White	**0.97 (0.97, 0.97)**	<0.01	**0.95 (0.91, 0.98)**	<0.01	**0.96 (0.96, 0.97)**	<0.01	**0.99 (0.98, 0.99)**	<0.01	**0.87 (0.84, 0.90)**	<0.01	**0.95 (0.94, 0.96)**	<0.01
Multiple	**0.95 (0.93, 0.97)**	<0.01	0.92 (0.69, 1.23)	0.57	**0.94 (0.92, 0.96)**	<0.01	0.99 (0.96, 1.03)	0.68	**0.72 (0.55, 0.94)**	0.02	0.94 (0.86, 1.02)	0.11
Unknown/Missing	1.00 (0.99, 1.01)	0.64	1.07 (0.96, 1.20)	0.23	0.99 (0.98, 1.00)	0.07	1.00 (0.99, 1.02)	0.64	1.01 (0.90, 1.13)	0.89	**1.07 (1.03, 1.11)**	<0.01
P for interaction	<0.01		0.22		<0.01		<0.01		0.01		<0.01	
**Ethnicity** [d]												
Hispanic	**0.98 (0.97, 0.98)**	<0.01	1.01 (0.93, 1.09)	0.88	**0.98 (0.97, 0.98)**	<0.01	**0.98 (0.97, 0.99)**	<0.01	**0.85 (0.78, 0.92)**	<0.01	1.00 (0.97, 1.02)	0.94
Non-Hispanic	**0.97 (0.97, 0.97)**	<0.01	**0.96 (0.93, 0.99)**	0.02	**0.97 (0.96, 0.97)**	<0.01	1.00 (0.99, 1.00)	0.14	**0.89 (0.86, 0.92)**	<0.01	**0.91 (0.90, 0.93)**	<0.01
P for interaction	<0.01		0.61		<0.01		0.01		0.11		<0.01	

Abbreviations: SDB=Sleep disordered breathing, CSA=Central Sleep Apnea, OSA=Obstructive Sleep Apnea, OUSA=Other/unspecified Sleep Apnea, AI/AN=American Indian/Alaskan Native, BAA= Black/African American, NHPI=Native Hawaiian/Pacific Islander, EBM= Environmental Burden Module, PR=Prevalence ratios, CI=Confidence intervals.

Results from log-binomial models with reported outputs of exponentiated beta coefficients (prevalence ratios) and their 95% confidence intervals.

[a] Adjusted for sex, race, and ethnicity

[b] Adjusted for age category, race, and ethnicity

[c] Adjusted for age category, sex, and ethnicity

[d] Adjusted for age category, sex, and race

P for the interaction effect is the p-value of a likelihood ratio test comparing log-binomial models with and without an interaction term with the exposure.

Bolded values indicate statistical significance (P<0.05).

NE- Not estimated.

eTable 6. Subgroup cross-sectional associations of per 0.1-unit increase in Social Vulnerability Module (SVM) ranks with sleep-disordered breathing (SDB), central sleep apnea (CSA), obstructive sleep apnea (OSA), and other/unspecified sleep apnea (OUSA), multiple sleep apneas, and procedure-based cases

	**Any SDB**		**Types of SDB**									
			**CSA**		**OSA**		**OUSA**		**Multiple sleep apneas**		**Procedure-based cases**	
	PR (95% CI)	P-value	PR (95% CI)	P-value	PR (95% CI)	P-value	PR (95% CI)	P-value	PR (95% CI)	P-value	PR (95% CI)	P-value
**Age category** [a]												
18-34 years	**1.03 (1.02, 1.04)**	<0.01	0.97 (0.88, 1.07)	0.53	**1.03 (1.02, 1.04)**	<0.01	**1.02 (1.01, 1.03)**	<0.01	1.04 (0.90, 1.20)	0.56	**1.07 (1.04, 1.11)**	<0.01
35-49 years	**1.02 (1.02, 1.03)**	<0.01	1.01 (0.94, 1.09)	0.78	**1.03 (1.02, 1.03)**	<0.01	1.01 (1.00, 1.02)	0.15	**1.08 (1.01, 1.17)**	0.03	**1.06 (1.03, 1.09)**	<0.01
≥50 years	**1.01 (1.01, 1.02)**	<0.01	1.03 (0.99, 1.07)	0.19	**1.01 (1.01, 1.02)**	<0.01	1.00 (0.99, 1.01)	0.87	**1.05 (1.01, 1.09)**	0.01	**1.09 (1.07, 1.11)**	<0.01
P for interaction	<0.01		0.58		<0.01		0.02		0.76		0.07	
**Sex** [b]												
Women	**1.03 (1.03, 1.03)**	<0.01	**1.06 (1.00, 1.11)**	0.03	**1.03 (1.03, 1.04)**	<0.01	**1.02 (1.01, 1.02)**	<0.01	**1.07 (1.02, 1.12)**	<0.01	**1.09 (1.07, 1.10)**	<0.01
Men	**1.00 (1.00, 1.01)**	0.03	0.99 (0.94, 1.03)	0.54	1.00 (1.00, 1.01)	0.03	0.99 (0.99, 1.00)	0.05	**1.04 (1.00, 1.09)**	0.04	**1.07 (1.05, 1.10)**	<0.01
Unknown/Missing	1.02 (0.93, 1.12)	0.63	NE	-	1.03 (0.91, 1.16)	0.65	1.06 (0.89, 1.26)	0.52	1.11 (0.51, 2.42)	0.80	0.64 (0.32, 1.28)	0.21
P for interaction effect	<0.01		0.21		<0.01		<0.01		0.94		0.10	
**Race** [c]												

	Any SDB		Types of SDB									
			**CSA**		**OSA**		**OUSA**		**Multiple sleep apneas**		**Procedure-based cases**	
	PR (95% CI)	P-value	PR (95% CI)	P-value	PR (95% CI)	P-value	PR (95% CI)	P-value	PR (95% CI)	P-value	PR (95% CI)	P-value
AI/AN	1.02 (1.00, 1.04)	0.07	1.11 (0.82, 1.48)	0.50	**1.03 (1.00, 1.05)**	0.04	0.99 (0.95, 1.03)	0.55	1.06 (0.80, 1.41)	0.69	1.07 (0.97, 1.18)	0.19
Asian	0.99 (0.98, 1.01)	0.36	0.79 (0.62, 1.02)	0.07	**0.98 (0.96, 0.99)**	<0.01	**0.97 (0.95, 1.00)**	0.02	0.90 (0.74, 1.09)	0.28	**1.30 (1.23, 1.37)**	<0.01
BAA	**1.01 (1.01, 1.02)**	<0.01	1.10 (0.98, 1.24)	0.12	**1.02 (1.01, 1.02)**	<0.01	1.00 (0.99, 1.01)	0.71	0.99 (0.89, 1.11)	0.90	1.01 (0.98, 1.04)	0.67
NHPI	NE	-	NE	-	NE	-	NE	-	NE	-	NE	-
White	**1.02 (1.02, 1.03)**	<0.01	1.01 (0.97, 1.05)	0.70	**1.02 (1.02, 1.03)**	<0.01	**1.01 (1.00, 1.02)**	<0.01	**1.07 (1.04, 1.11)**	<0.01	**1.09 (1.07, 1.11)**	<0.01
Multiple	**1.02 (1.00, 1.04)**	0.03	1.20 (0.85, 1.70)	0.29	**1.03 (1.00, 1.06)**	0.03	1.01 (0.98, 1.05)	0.49	0.87 (0.70, 1.08)	0.21	1.00 (0.92, 1.09)	0.95
Unknown/Missing	1.01 (1.00, 1.01)	0.13	1.06 (0.94, 1.20)	0.31	1.00 (1.00, 1.01)	0.36	1.00 (0.99, 1.01)	0.97	1.06 (0.93, 1.20)	0.4	**1.06 (1.02, 1.10)**	<0.01
P for interaction	0.02		0.19		<0.01		0.24		0.34		<0.01	
**Ethnicity** [d]												
Hispanic	**0.98 (0.98, 0.99)**	<0.01	1.04 (0.94, 1.15)	0.45	**0.99 (0.98, 0.99)**	<0.01	**0.97 (0.96, 0.98)**	<0.01	1.03 (0.94, 1.13)	0.53	**0.96 (0.93, 0.98)**	<0.01
Non-Hispanic	**1.03 (1.03, 1.03)**	<0.01	1.01 (0.98, 1.05)	0.49	**1.03 (1.03, 1.03)**	<0.01	**1.02 (1.01, 1.02)**	<0.01	**1.07 (1.03, 1.11)**	<0.01	**1.12 (1.10, 1.14)**	<0.01
P for interaction	<0.01		0.87		<0.01		<0.01		0.43		<0.01	

Abbreviations: SDB=Sleep disordered breathing, CSA=Central Sleep Apnea, OSA=Obstructive Sleep Apnea, OUSA=Other/unspecified Sleep Apnea, AI/AN=American Indian/Alaskan Native, BAA= Black/African American, NHPI=Native Hawaiian/Pacific Islander, SVM= Social Vulnerability Module, PR=Prevalence ratios, CI=Confidence intervals.

Results from log-binomial models with reported outputs of exponentiated beta coefficients (prevalence ratios) and their 95% confidence intervals.

[a] Adjusted for sex, race, and ethnicity

[b] Adjusted for age category, race, and ethnicity

[c] Adjusted for age category, sex, and ethnicity

[d] Adjusted for age category, sex, and race

P for the interaction effect is the p-value of a likelihood ratio test comparing log-binomial models with and without an interaction term with the exposure.

Bolded values indicate statistical significance (P<0.05).

NE- Not estimated.

eTable 7. Subgroup cross-sectional associations of per 0.1-unit increase in Health Vulnerability Module (HVM) ranks with sleep-disordered breathing (SDB), central sleep apnea (CSA), obstructive sleep apnea (OSA), and other/unspecified sleep apnea (OUSA), multiple sleep apneas, and procedure-based cases

	**Any SDB**		**Types of SDB**									
			**CSA**		**OSA**		**OUSA**		**Multiple sleep apneas**		**Procedure-based cases**	
	PR (95% CI)	P-value	PR (95% CI)	P-value	PR (95% CI)	P-value	PR (95% CI)	P-value	PR (95% CI)	P-value	PR (95% CI)	P-value
**Age category** [a]												
18-34 years	**1.04 (1.03, 1.04)**	<0.01	1.02 (0.94, 1.12)	0.60	**1.03 (1.03, 1.04)**	<0.01	**1.04 (1.03, 1.05)**	<0.01	1.01 (0.89, 1.15)	0.84	**1.07 (1.04, 1.10)**	<0.01
35-49 years	**1.03 (1.03, 1.04)**	<0.01	1.03 (0.97, 1.10)	0.31	**1.03 (1.03, 1.04)**	<0.01	**1.03 (1.02, 1.04)**	<0.01	1.05 (0.99, 1.12)	0.10	**1.05 (1.03, 1.08)**	<0.01
≥50 years	**1.02 (1.01, 1.02)**	<0.01	1.03 (1.00, 1.07)	0.07	**1.01 (1.01, 1.02)**	<0.01	**1.02 (1.01, 1.02)**	<0.01	**1.03 (1.00, 1.06)**	0.03	**1.03 (1.02, 1.04)**	<0.01
P for interaction	<0.01		0.94		<0.01		<0.01		0.97		0.10	
**Sex** [b]												
Women	**1.03 (1.02, 1.03)**	<0.01	1.04 (1.00, 1.09)	0.05	**1.02 (1.02, 1.03)**	<0.01	**1.03 (1.02, 1.04)**	<0.01	1.03 (0.99, 1.07)	0.14	**1.04 (1.03, 1.05)**	<0.01
Men	**1.02 (1.01, 1.02)**	<0.01	1.02 (0.98, 1.06)	0.31	**1.02 (1.01, 1.02)**	<0.01	**1.02 (1.01, 1.02)**	<0.01	**1.04 (1.01, 1.08)**	0.02	**1.04 (1.02, 1.06)**	<0.01
Unknown/Missing	0.95 (0.85, 1.06)	0.38	NE	-	0.93 (0.81, 1.07)	0.33	1.05 (0.88, 1.26)	0.58	0.00 (0.00, Inf)	1.00	0.70 (0.33, 1.47)	0.35
P for interaction effect	<0.01		0.66		<0.01		<0.01		0.39		0.66	
**Race** [c]												

	Any SDB		Types of SDB									
			CSA		OSA		OUSA		Multiple sleep apneas		Procedure-based cases	
	PR (95% CI)	P-value	PR (95% CI)	P-value	PR (95% CI)	P-value	PR (95% CI)	P-value	PR (95% CI)	P-value	PR (95% CI)	P-value
AI/AN	**1.05 (1.04, 1.07)**	<0.01	1.14 (0.92, 1.41)	0.23	**1.07 (1.05, 1.09)**	<0.01	1.01 (0.98, 1.05)	0.47	1.04 (0.84, 1.29)	0.72	1.03 (0.95, 1.11)	0.49
Asian	1.01 (1.00, 1.03)	0.07	0.94 (0.68, 1.29)	0.68	0.98 (0.97, 1.00)	0.09	0.99 (0.97, 1.02)	0.68	0.99 (0.78, 1.25)	0.92	**1.29 (1.23, 1.34)**	<0.01
BAA	**1.01 (1.01, 1.02)**	<0.01	1.01 (0.93, 1.09)	0.81	**1.01 (1.01, 1.02)**	<0.01	**1.02 (1.01, 1.03)**	<0.01	1.06 (0.97, 1.16)	0.18	0.99 (0.97, 1.01)	0.38
NHPI	NE	-	NE	-	NE	-	NE	-	NE	-	NE	-
White	**1.02 (1.02, 1.03)**	<0.01	1.02 (0.99, 1.06)	0.16	**1.02 (1.02, 1.02)**	<0.01	**1.03 (1.02, 1.03)**	<0.01	**1.03 (1.00, 1.06)**	0.04	**1.04 (1.02, 1.05)**	<0.01
Multiple	**1.04 (1.02, 1.06)**	<0.01	1.15 (0.89, 1.49)	0.28	**1.04 (1.01, 1.06)**	<0.01	**1.05 (1.01, 1.09)**	<0.01	1.06 (0.87, 1.29)	0.57	1.06 (0.98, 1.15)	0.13
Unknown/Missing	**1.02 (1.01, 1.02)**	<0.01	1.11 (1.00, 1.23)	0.05	**1.01 (1.00, 1.02)**	<0.01	**1.02 (1.01, 1.03)**	<0.01	1.08 (0.97, 1.20)	0.18	**1.05 (1.02, 1.09)**	<0.01
P for interaction	<0.01		0.60		<0.01		0.20		0.99		<0.01	
**Ethnicity** [d]												
Hispanic	1.00 (1.00, 1.01)	0.14	1.07 (0.99, 1.17)	0.09	1.00 (1.00, 1.01)	0.14	1.00 (0.99, 1.01)	1.00	1.04 (0.96, 1.13)	0.29	1.01 (0.98, 1.03)	0.58
Non-Hispanic	**1.03 (1.02, 1.03)**	<0.01	1.02 (0.99, 1.05)	0.27	**1.02 (1.02, 1.03)**	<0.01	**1.03 (1.02, 1.03)**	<0.01	**1.03 (1.00, 1.06)**	0.03	**1.05 (1.04, 1.06)**	<0.01
P for interaction	<0.01		0.19		<0.01		<0.01		0.90		<0.01	

Abbreviations: SDB=Sleep disordered breathing, CSA=Central Sleep Apnea, OSA=Obstructive Sleep Apnea, OUSA=Other/unspecified Sleep Apnea, AI/AN=American Indian/Alaskan Native, BAA= Black/African American, NHPI=Native Hawaiian/Pacific Islander, HVM=Health Vulnerability Module, PR=Prevalence ratios, CI=Confidence intervals.

Results from log-binomial models with reported outputs of exponentiated beta coefficients (prevalence ratios) and their 95% confidence intervals.

[a] Adjusted for sex, race, and ethnicity

[b] Adjusted for age category, race, and ethnicity

[c] Adjusted for age category, sex, and ethnicity

[d] Adjusted for age category, sex, and race

P for the interaction effect is the p-value of a likelihood ratio test comparing log-binomial models with and without an interaction term with the exposure.

Bolded values indicate statistical significance (P<0.05).

NE- Not estimated.

eTable 8. Sensitivity analysis for subgroup cross-sectional associations of per 0.1-unit increase in Environmental, Social, and Health Burden (ESHB) ranks with sleep-disordered breathing (SDB), central sleep apnea (CSA), obstructive sleep apnea (OSA), and other/unspecified sleep apnea (OUSA), multiple sleep apneas, and procedure-based cases

	**Any SDB**		**Types of SDB**									
			**CSA**		**OSA**		**OUSA**		**Multiple sleep apneas**		**Procedure-based cases**	
	PR (Bonferroni-corrected 95% CI)	P-value	PR (Bonferroni-corrected 95% CI)	P-value	PR (Bonferroni-corrected 95% CI)	P-value	PR (Bonferroni-corrected 95% CI)	P-value	PR (Bonferroni-corrected 95% CI)	P-value	PR (Bonferroni-corrected 95% CI)	P-value
**Age category** [a]												
18-34 years	**1.02 (1.01, 1.03)**	<0.01	1.00 (0.88, 1.12)	0.94	**1.02 (1.01, 1.03)**	<0.01	**1.02 (1.01, 1.04)**	<0.01	0.94 (0.80, 1.10)	0.35	**1.07 (1.03, 1.12)**	<0.01
35-49 years	**1.02 (1.01, 1.02)**	<0.01	1.02 (0.94, 1.11)	0.57	**1.02 (1.01, 1.02)**	<0.01	**1.01 (1.00, 1.02)**	<0.01	1.02 (0.94, 1.11)	0.54	**1.03 (1.00, 1.07)**	0.01
≥50 years	1.00 (1.00, 1.01)	0.17	1.01 (0.96, 1.05)	0.79	1.00 (0.99, 1.00)	0.28	1.01 (1.00, 1.01)	0.06	0.99 (0.95, 1.03)	0.64	**1.04 (1.03, 1.06)**	<0.01
P for interaction	<0.01		0.94		<0.01		0.05		0.74		0.09	
**Sex** [b]												
Women	**1.02 (1.02, 1.02)**	<0.01	1.04 (0.98, 1.10)	0.16	**1.02 (1.01, 1.02)**	<0.01	**1.02 (1.02, 1.03)**	<0.01	1.00 (0.94, 1.05)	0.89	**1.05 (1.03, 1.07)**	<0.01
Men	**1.00 (0.99, 1.00)**	<0.01	0.98 (0.93, 1.04)	0.48	**0.99 (0.99, 1.00)**	<0.01	1.00 (0.99, 1.01)	0.51	0.99 (0.95, 1.04)	0.75	**1.04 (1.02, 1.07)**	<0.01
Unknown/Missing	0.99 (0.88, 1.12)	0.85	NE	-	0.99 (0.85, 1.15)	0.86	1.04 (0.83, 1.30)	0.70	1.09 (0.42, 2.82)	0.83	0.70 (0.33, 1.47)	0.25
P for interaction effect	<0.01		0.40		<0.01		<0.01		0.87		0.29	

	**Any SDB**		**Types of SDB**									
			**CSA**		**OSA**		**OUSA**		**Multiple sleep apneas**		**Procedure-based cases**	
	PR (Bonferroni-corrected 95% CI)	P-value	PR (Bonferroni-corrected 95% CI)	P-value	PR (Bonferroni-corrected 95% CI)	P-value	PR (Bonferroni-corrected 95% CI)	P-value	PR (Bonferroni-corrected 95% CI)	P-value	PR (Bonferroni-corrected 95% CI)	P-value
**Race** [c]												
AI/AN	**1.02 (0.99, 1.05)**	0.04	1.19 (0.79, 1.77)	0.25	**1.03 (1.00, 1.06)**	0.02	0.98 (0.93, 1.04)	0.43	1.07 (0.74, 1.54)	0.63	1.05 (0.92, 1.20)	0.28
Asian	0.99 (0.97, 1.01)	0.10	0.87 (0.61, 1.23)	0.27	**0.97 (0.95, 0.99)**	<0.01	0.98 (0.95, 1.01)	0.13	0.89 (0.67, 1.18)	0.26	**1.23 (1.15, 1.31)**	<0.01
BAA	**1.01 (1.00, 1.01)**	0.02	1.05 (0.91, 1.21)	0.34	1.01 (1.00, 1.02)	0.08	**1.02 (1.00, 1.03)**	<0.01	1.04 (0.89, 1.21)	0.49	**0.95 (0.92, 0.99)**	<0.01
NHPI	NE	-	NE	-	NE	-	NE	-	NE	-	NE	-
White	**1.01 (1.01, 1.01)**	<0.01	0.99 (0.94, 1.04)	0.69	**1.01 (1.00, 1.01)**	<0.01	**1.01 (1.01, 1.02)**	<0.01	0.99 (0.95, 1.04)	0.67	**1.05 (1.03, 1.07)**	<0.01
Multiple	1.01 (0.98, 1.04)	0.35	1.12 (0.74, 1.71)	0.45	1.00 (0.97, 1.04)	0.73	1.02 (0.97, 1.08)	0.20	0.85 (0.63, 1.15)	0.15	1.01 (0.90, 1.13)	0.81
Unknown/Missing	**1.01 (1.00, 1.02)**	<0.01	1.10 (0.94, 1.30)	0.10	1.00 (0.99, 1.02)	0.27	1.01 (0.99, 1.03)	0.13	1.07 (0.90, 1.26)	0.30	**1.11 (1.05, 1.17)**	<0.01
P for interaction	0.01		0.25		<0.01		0.28		0.48		<0.01	
**Ethnicity** [d]												
Hispanic	**0.98 (0.98, 0.99)**	<0.01	1.06 (0.95, 1.17)	0.24	**0.99 (0.98, 0.99)**	<0.01	**0.98 (0.97, 0.99)**	<0.01	0.96 (0.87, 1.05)	0.31	1.00 (0.97, 1.03)	0.90

	Any SDB		Types of SDB									
			CSA		OSA		OUSA		Multiple sleep apneas		Procedure-based cases	
	PR (Bonferroni-corrected 95% CI)	P-value	PR (Bonferroni-corrected 95% CI)	P-value	PR (Bonferroni-corrected 95% CI)	P-value	PR (Bonferroni-corrected 95% CI)	P-value	PR (Bonferroni-corrected 95% CI)	P-value	PR (Bonferroni-corrected 95% CI)	P-value
Non-Hispanic	**1.02 (1.01, 1.02)**	<0.01	1.00 (0.96, 1.04)	0.89	**1.01 (1.01, 1.02)**	<0.01	**1.02 (1.02, 1.03)**	<0.01	1.00 (0.96, 1.04)	0.94	**1.06 (1.04, 1.07)**	<0.01
P for interaction	<0.01		0.45		<0.01		<0.01		0.56		<0.01	

Abbreviations: SDB=Sleep disordered breathing, CSA=Central Sleep Apnea, OSA=Obstructive Sleep Apnea, OUSA=Other/unspecified Sleep Apnea, AI/AN=American Indian/Alaskan Native, BAA= Black/African American, NHPI=Native Hawaiian/Pacific Islander, ESHB=Environmental, Social, and Health Burden, PR=Prevalence ratios, CI=Confidence intervals.

Results from log-binomial models with reported outputs of exponentiated beta coefficients (prevalence ratios) and their Bonferroni-corrected 95% confidence intervals.

[a] Adjusted for sex, race, and ethnicity (Bonferroni-corrected p-critical value = 0.017).

[b] Adjusted for age category, race, and ethnicity (Bonferroni-corrected p-critical value = 0.017).

[c] Adjusted for age category, sex, and ethnicity (Bonferroni-corrected p-critical value = 0.008).

[d] Adjusted for age category, sex, and race (Bonferroni-corrected p-critical value = 0.025).

P for the interaction effect are the p-value of a likelihood ratio test comparing log-binomial models with and without an interaction term.

Bolded values indicate statistical significance (P<0.05).

NE- Not estimated.

eTable 9. Sensitivity analysis for subgroup cross-sectional associations of per 0.1-unit increase in Environmental Burden Module (EBM) ranks with sleep-disordered breathing (SDB), central sleep apnea (CSA), obstructive sleep apnea (OSA), and other/unspecified sleep apnea (OUSA), multiple sleep apneas, and procedure-based cases

	**Any SDB**		**Types of SDB**									
			**CSA**		**OSA**		**OUSA**		**Multiple sleep apneas**		**Procedure-based cases**	
	PR (Bonferroni-corrected 95% CI)	P-value	PR (Bonferroni-corrected 95% CI)	P-value	PR (Bonferroni-corrected 95% CI)	P-value	PR (Bonferroni-corrected 95% CI)	P-value	PR (Bonferroni-corrected 95% CI)	P-value	PR (Bonferroni-corrected 95% CI)	P-value
**Age category** [a]												
18-34 years	**0.98 (0.97, 0.99)**	<0.01	1.01 (0.90, 1.13)	0.82	**0.97 (0.96, 0.99)**	<0.01	0.99 (0.98, 1.00)	0.06	**0.80 (0.67, 0.96)**	<0.01	**0.97 (0.93, 1.00)**	0.03
35-49 years	**0.97 (0.97, 0.98)**	<0.01	0.98 (0.90, 1.07)	0.57	**0.97 (0.97, 0.98)**	<0.01	**0.99 (0.98, 1.00)**	0.01	**0.90 (0.83, 0.98)**	<0.01	**0.93 (0.90, 0.96)**	<0.01
≥50 years	**0.97 (0.97, 0.97)**	<0.01	**0.95 (0.91, 1.00)**	0.01	**0.96 (0.96, 0.97)**	<0.01	**0.99 (0.98, 1.00)**	<0.01	**0.89 (0.85, 0.93)**	<0.01	**0.94 (0.93, 0.96)**	<0.01
P for interaction	<0.01		0.47		<0.01		0.96		0.48		0.06	
**Sex** [b]												
Women	**0.98 (0.98, 0.98)**	<0.01	0.97 (0.92, 1.03)	0.19	**0.98 (0.97, 0.98)**	<0.01	1.00 (0.99, 1.01)	0.93	**0.89 (0.84, 0.94)**	<0.01	**0.94 (0.93, 0.96)**	<0.01
Men	**0.96 (0.96, 0.97)**	<0.01	0.96 (0.91, 1.01)	0.07	**0.96 (0.95, 0.96)**	<0.01	**0.98 (0.97, 0.99)**	<0.01	**0.88 (0.84, 0.93)**	<0.01	**0.94 (0.91, 0.96)**	<0.01
Unknown/Missing	0.98 (0.88, 1.10)	0.73	NE	-	0.98 (0.85, 1.13)	0.74	0.99 (0.80, 1.22)	0.87	1.43 (0.52, 3.91)	0.40	0.84 (0.44, 1.61)	0.52
P for interaction effect	<0.01		1.00		<0.01		<0.01		0.43		0.54	

	Any SDB		Types of SDB									
			CSA		OSA		OUSA		Multiple sleep apneas		Procedure-based cases	
	PR (Bonferroni-corrected 95% CI)	P-value	PR (Bonferroni-corrected 95% CI)	P-value	PR (Bonferroni-corrected 95% CI)	P-value	PR (Bonferroni-corrected 95% CI)	P-value	PR (Bonferroni-corrected 95% CI)	P-value	PR (Bonferroni-corrected 95% CI)	P-value
**Race** [c]												
AI/AN	**0.94 (0.92, 0.97)**	<0.01	1.04 (0.73, 1.49)	0.77	**0.93 (0.90, 0.96)**	<0.01	0.97 (0.92, 1.02)	0.12	0.97 (0.68, 1.39)	0.82	0.97 (0.86, 1.10)	0.55
Asian	**0.97 (0.95, 0.98)**	<0.01	1.02 (0.72, 1.44)	0.89	**0.97 (0.95, 0.99)**	<0.01	0.99 (0.95, 1.02)	0.26	0.88 (0.66, 1.18)	0.25	**0.90 (0.84, 0.96)**	<0.01
BAA	**0.98 (0.98, 0.99)**	<0.01	1.05 (0.91, 1.20)	0.38	**0.98 (0.97, 0.99)**	<0.01	1.01 (0.99, 1.02)	0.17	1.01 (0.87, 1.17)	0.86	**0.86 (0.82, 0.89)**	<0.01
NHPI	NE	-	NE	-	NE	-	NE	-	NE	-	NE	-
White	**0.97 (0.97, 0.97)**	<0.01	**0.95 (0.90, 0.99)**	<0.01	**0.96 (0.96, 0.97)**	<0.01	**0.99 (0.98, 0.99)**	<0.01	**0.87 (0.83, 0.91)**	<0.01	**0.95 (0.93, 0.97)**	<0.01
Multiple	**0.95 (0.93, 0.98)**	<0.01	0.92 (0.61, 1.37)	0.57	**0.94 (0.91, 0.97)**	<0.01	0.99 (0.94, 1.04)	0.68	**0.72 (0.49, 1.04)**	0.02	0.94 (0.84, 1.05)	0.11
Unknown/Missing	1.00 (0.99, 1.01)	0.64	1.07 (0.92, 1.25)	0.23	0.99 (0.98, 1.00)	0.07	1.00 (0.98, 1.02)	0.64	1.01 (0.86, 1.18)	0.89	**1.07 (1.01, 1.12)**	<0.01
P for interaction	<0.01		0.22		<0.01		<0.01		0.01		<0.01	
**Ethnicity** [d]												
Hispanic	**0.98 (0.97, 0.98)**	<0.01	1.01 (0.91, 1.11)	0.88	**0.98 (0.97, 0.98)**	<0.01	**0.98 (0.97, 0.99)**	<0.01	**0.85 (0.77, 0.93)**	<0.01	1.00 (0.97, 1.03)	0.94

	Any SDB		Types of SDB									
			CSA		OSA		OUSA		Multiple sleep apneas		Procedure-based cases	
	PR (Bonferroni-corrected 95% CI)	P-value	PR (Bonferroni-corrected 95% CI)	P-value	PR (Bonferroni-corrected 95% CI)	P-value	PR (Bonferroni-corrected 95% CI)	P-value	PR (Bonferroni-corrected 95% CI)	P-value	PR (Bonferroni-corrected 95% CI)	P-value
Non-Hispanic	**0.97 (0.97, 0.97)**	<0.01	**0.96 (0.92, 1.00)**	0.02	**0.97 (0.96, 0.97)**	<0.01	1.00 (0.99, 1.00)	0.14	**0.89 (0.85, 0.92)**	<0.01	**0.91 (0.90, 0.93)**	<0.01
P for interaction	<0.01		0.61		<0.01		0.01		0.11		<0.01	

Abbreviations: SDB=Sleep disordered breathing, CSA=Central Sleep Apnea, OSA=Obstructive Sleep Apnea, OUSA=Other/unspecified Sleep Apnea, AI/AN=American Indian/Alaskan Native, BAA= Black/African American, NHPI=Native Hawaiian/Pacific Islander, EBM= Environmental Burden Module, PR=Prevalence ratios, CI=Confidence intervals.

Results from log-binomial models with reported outputs of exponentiated beta coefficients (prevalence ratios) and their Bonferroni-corrected 95% confidence intervals.

[a] Adjusted for sex, race, and ethnicity (Bonferroni-corrected p-critical value = 0.017).

[b] Adjusted for age category, race, and ethnicity (Bonferroni-corrected p-critical value = 0.017).

[c] Adjusted for age category, sex, and ethnicity (Bonferroni-corrected p-critical value = 0.008).

[d] Adjusted for age category, sex, and race (Bonferroni-corrected p-critical value = 0.025).

P for the interaction effect is the p-value of a likelihood ratio test comparing log-binomial models with and without an interaction term.

Bolded values indicate statistical significance (P<0.05).

NE- Not estimated.

eTable 10. Sensitivity analysis for subgroup cross-sectional associations of per 0.1-unit increase in Social Vulnerability Module (SVM) ranks with sleep-disordered breathing (SDB), central sleep apnea (CSA), obstructive sleep apnea (OSA), and other/unspecified sleep apnea (OUSA), multiple sleep apneas, and procedure-based cases

	**Any SDB**		**Types of SDB**									
			**CSA**		**OSA**		**OUSA**		**Multiple sleep apneas**		**Procedure-based cases**	
	PR (Bonferroni-corrected 95% CI)	P-value	PR (Bonferroni-corrected 95% CI)	P-value	PR (Bonferroni-corrected 95% CI)	P-value	PR (Bonferroni-corrected 95% CI)	P-value	PR (Bonferroni-corrected 95% CI)	P-value	PR (Bonferroni-corrected 95% CI)	P-value
**Age category** [a]												
18-34 years	**1.03 (1.02, 1.04)**	<0.01	0.97 (0.86, 1.09)	0.53	**1.03 (1.02, 1.04)**	<0.01	**1.02 (1.00, 1.04)**	<0.01	1.04 (0.88, 1.24)	0.56	**1.07 (1.03, 1.12)**	<0.01
35-49 years	**1.02 (1.02, 1.03)**	<0.01	1.01 (0.92, 1.11)	0.78	**1.03 (1.02, 1.04)**	<0.01	1.01 (1.00, 1.02)	0.15	**1.08 (0.99, 1.19)**	0.03	**1.06 (1.02, 1.10)**	<0.01
≥50 years	**1.01 (1.01, 1.02)**	<0.01	1.03 (0.98, 1.08)	0.19	**1.01 (1.01, 1.02)**	<0.01	1.00 (0.99, 1.01)	0.87	**1.05 (1.00, 1.10)**	0.01	**1.09 (1.07, 1.11)**	<0.01
P for interaction	<0.01		0.58		<0.01		0.02		0.76		0.07	
**Sex** [b]												
Women	**1.03 (1.03, 1.03)**	<0.01	**1.06 (0.99, 1.13)**	0.03	**1.03 (1.03, 1.04)**	<0.01	**1.02 (1.01, 1.02)**	<0.01	**1.07 (1.01, 1.14)**	<0.01	**1.09 (1.07, 1.11)**	<0.01
Men	**1.00 (1.00, 1.01)**	0.03	0.99 (0.94, 1.04)	0.54	**1.00 (1.00, 1.01)**	0.03	0.99 (0.99, 1.00)	0.05	**1.04 (0.99, 1.10)**	0.04	**1.07 (1.05, 1.10)**	<0.01
Unknown/Missing	1.02 (0.91, 1.15)	0.63	NE	-	1.03 (0.89, 1.19)	0.65	1.06 (0.86, 1.31)	0.52	1.11 (0.43, 2.88)	0.80	0.64 (0.27, 1.50)	0.21
P for interaction effect	<0.01		0.21		<0.01		<0.01		0.94		0.10	

	Any SDB		Types of SDB									
			CSA		OSA		OUSA		Multiple sleep apneas		Procedure-based cases	
	PR (Bonferroni-corrected 95% CI)	P-value	PR (Bonferroni-corrected 95% CI)	P-value	PR (Bonferroni-corrected 95% CI)	P-value	PR (Bonferroni-corrected 95% CI)	P-value	PR (Bonferroni-corrected 95% CI)	P-value	PR (Bonferroni-corrected 95% CI)	P-value
**Race** [c]												
AI/AN	1.02 (0.99, 1.05)	0.07	1.11 (0.74, 1.65)	0.50	**1.03 (0.99, 1.06)**	0.04	0.99 (0.93, 1.05)	0.55	1.06 (0.72, 1.56)	0.69	1.07 (0.93, 1.23)	0.19
Asian	0.99 (0.98, 1.01)	0.36	0.79 (0.56, 1.12)	0.07	**0.98 (0.96, 1.00)**	<0.01	**0.97 (0.94, 1.01)**	0.02	0.90 (0.68, 1.17)	0.28	**1.30 (1.21, 1.40)**	<0.01
BAA	**1.01 (1.00, 1.02)**	<0.01	1.10 (0.93, 1.29)	0.12	**1.02 (1.01, 1.03)**	<0.01	1.00 (0.99, 1.02)	0.71	0.99 (0.85, 1.16)	0.90	1.01 (0.96, 1.05)	0.67
NHPI	NE	-	NE	-	NE	-	NE	-	NE	-	NE	-
White	**1.02 (1.02, 1.03)**	<0.01	1.01 (0.96, 1.06)	0.70	**1.02 (1.02, 1.03)**	<0.01	**1.01 (1.00, 1.02)**	<0.01	**1.07 (1.02, 1.13)**	<0.01	**1.09 (1.07, 1.11)**	<0.01
Multiple	1.02 (0.99, 1.05)	0.03	1.20 (0.75, 1.94)	0.29	**1.03 (0.99, 1.07)**	0.03	1.01 (0.96, 1.07)	0.49	0.87 (0.65, 1.17)	0.21	1.00 (0.89, 1.13)	0.95
Unknown/Missing	1.01 (1.00, 1.02)	0.13	1.06 (0.90, 1.26)	0.31	1.00 (0.99, 1.02)	0.36	1.00 (0.98, 1.02)	0.97	1.06 (0.89, 1.26)	0.40	**1.06 (1.01, 1.12)**	<0.01
P for interaction	0.02		0.19		<0.01		0.24		0.34		<0.01	
**Ethnicity** [d]												
Hispanic	**0.98 (0.97, 0.99)**	<0.01	1.04 (0.93, 1.16)	0.45	**0.99 (0.98, 0.99)**	<0.01	**0.97 (0.96, 0.98)**	<0.01	1.03 (0.92, 1.15)	0.53	**0.96 (0.93, 0.99)**	<0.01

	Any SDB		Types of SDB									
			CSA		OSA		OUSA		Multiple sleep apneas		Procedure-based cases	
	PR (Bonferroni-corrected 95% CI)	P-value	PR (Bonferroni-corrected 95% CI)	P-value	PR (Bonferroni-corrected 95% CI)	P-value	PR (Bonferroni-corrected 95% CI)	P-value	PR (Bonferroni-corrected 95% CI)	P-value	PR (Bonferroni-corrected 95% CI)	P-value
Non-Hispanic	**1.03 (1.03, 1.03)**	<0.01	1.01 (0.97, 1.06)	0.49	**1.03 (1.02, 1.03)**	<0.01	**1.02 (1.01, 1.02)**	<0.01	**1.07 (1.02, 1.11)**	<0.01	**1.12 (1.10, 1.14)**	<0.01
P for interaction	<0.01		0.19		<0.01		0.24		0.34		<0.01	

Abbreviations: SDB=Sleep disordered breathing, CSA=Central Sleep Apnea, OSA=Obstructive Sleep Apnea, OUSA=Other/unspecified Sleep Apnea, AI/AN=American Indian/Alaskan Native, BAA= Black/African American, NHPI=Native Hawaiian/Pacific Islander, SVM= Social Vulnerability Module, PR=Prevalence ratios, CI=Confidence intervals.

Results from log-binomial models with reported outputs of exponentiated beta coefficients (prevalence ratios) and their Bonferroni-corrected 95% confidence intervals.

[a] Adjusted for sex, race, and ethnicity (Bonferroni-corrected p-critical value = 0.017).

[b] Adjusted for age category, race, and ethnicity (Bonferroni-corrected p-critical value = 0.017).

[c] Adjusted for age category, sex, and ethnicity (Bonferroni-corrected p-critical value = 0.008).

[d] Adjusted for age category, sex, and race (Bonferroni-corrected p-critical value = 0.025).

P for the interaction effect is the p-value of a likelihood ratio test comparing log-binomial models with and without an interaction term.

Bolded values indicate statistical significance (P<0.05).

NE- Not estimated.

eTable 11. Sensitivity analysis for subgroup cross-sectional associations of per 0.1-unit increase in Health Vulnerability Module (HVM) ranks with sleep-disordered breathing (SDB), central sleep apnea (CSA), obstructive sleep apnea (OSA), and other/unspecified sleep apnea (OUSA), multiple sleep apneas, and procedure-based cases

	**Any SDB**		**Types of SDB**									
			**CSA**		**OSA**		**OUSA**		**Multiple sleep apneas**		**Procedure-based cases**	
	PR (Bonferroni-corrected 95% CI)	P-value	PR (Bonferroni-corrected 95% CI)	P-value	PR (Bonferroni-corrected 95% CI)	P-value	PR (Bonferroni-corrected 95% CI)	P-value	PR (Bonferroni-corrected 95% CI)	P-value	PR (Bonferroni-corrected 95% CI)	P-value
**Age category** [a]												
18-34 years	**1.04 (1.03, 1.05)**	<0.01	1.02 (0.92, 1.14)	0.60	**1.03 (1.02, 1.05)**	<0.01	**1.04 (1.02, 1.05)**	<0.01	1.01 (0.87, 1.18)	0.84	**1.07 (1.03, 1.11)**	<0.01
35-49 years	**1.03 (1.03, 1.04)**	<0.01	1.03 (0.96, 1.12)	0.31	**1.03 (1.03, 1.04)**	<0.01	**1.03 (1.02, 1.04)**	<0.01	1.05 (0.98, 1.13)	0.1	**1.05 (1.02, 1.08)**	<0.01
≥50 years	**1.02 (1.01, 1.02)**	<0.01	1.03 (0.99, 1.07)	0.07	**1.01 (1.01, 1.02)**	<0.01	**1.02 (1.01, 1.02)**	<0.01	**1.03 (1.00, 1.07)**	0.03	**1.03 (1.02, 1.05)**	<0.01
P for interaction	<0.01		0.94		<0.01		<0.01		0.97		0.10	
**Sex** [b]												
Women	**1.03 (1.02, 1.03)**	<0.01	1.04 (0.99, 1.10)	0.05	**1.02 (1.02, 1.03)**	<0.01	**1.03 (1.02, 1.04)**	<0.01	1.03 (0.98, 1.08)	0.14	**1.04 (1.02, 1.06)**	<0.01
Men	**1.02 (1.01, 1.02)**	<0.01	1.02 (0.97, 1.07)	0.31	**1.02 (1.01, 1.02)**	<0.01	**1.02 (1.01, 1.02)**	<0.01	**1.04 (1.00, 1.09)**	0.02	**1.04 (1.02, 1.06)**	<0.01
Unknown/Missing	0.95 (0.83, 1.09)	0.38	NE	-	0.93 (0.78, 1.11)	0.33	1.05 (0.84, 1.32)	0.58	0.00 (0.00, Inf)	1.00	0.70 (0.28, 1.73)	0.35
P for interaction effect	<0.01		0.66		<0.01		<0.01		0.39		0.66	

	Any SDB		Types of SDB									
			CSA		OSA		OUSA		Multiple sleep apneas		Procedure-based cases	
	PR (Bonferroni-corrected 95% CI)	P-value	PR (Bonferroni-corrected 95% CI)	P-value	PR (Bonferroni-corrected 95% CI)	P-value	PR (Bonferroni-corrected 95% CI)	P-value	PR (Bonferroni-corrected 95% CI)	P-value	PR (Bonferroni-corrected 95% CI)	P-value
**Race** [c]												
AI/AN	**1.05 (1.03, 1.08)**	<0.01	1.14 (0.85, 1.53)	0.23	**1.07 (1.04, 1.10)**	<0.01	1.01 (0.97, 1.06)	0.47	1.04 (0.77, 1.41)	0.72	1.03 (0.92, 1.15)	0.49
Asian	1.01 (0.99, 1.03)	0.07	0.94 (0.60, 1.45)	0.68	0.98 (0.96, 1.01)	0.09	0.99 (0.96, 1.03)	0.68	0.99 (0.71, 1.37)	0.92	**1.29 (1.21, 1.36)**	<0.01
BAA	**1.01 (1.01, 1.02)**	<0.01	1.01 (0.90, 1.13)	0.81	**1.01 (1.00, 1.02)**	<0.01	**1.02 (1.01, 1.03)**	<0.01	1.06 (0.94, 1.20)	0.18	0.99 (0.96, 1.02)	0.38
NHPI	NE	-	NE	-	NE	-	NE	-	NE	-	NE	-
White	**1.02 (1.02, 1.03)**	<0.01	1.02 (0.98, 1.07)	0.16	**1.02 (1.02, 1.03)**	<0.01	**1.03 (1.02, 1.03)**	<0.01	**1.03 (0.99, 1.07)**	0.04	**1.04 (1.02, 1.05)**	<0.01
Multiple	**1.04 (1.02, 1.07)**	<0.01	1.15 (0.81, 1.63)	0.28	**1.04 (1.00, 1.07)**	<0.01	**1.05 (1.00, 1.10)**	<0.01	1.06 (0.81, 1.39)	0.57	1.06 (0.95, 1.18)	0.13
Unknown/Missing	**1.02 (1.01, 1.03)**	<0.01	1.11 (0.96, 1.27)	0.05	**1.01 (1.00, 1.03)**	<0.01	**1.02 (1.00, 1.04)**	<0.01	1.08 (0.93, 1.25)	0.18	**1.05 (1.01, 1.11)**	<0.01
P for interaction	<0.01		0.60		<0.01		0.20		0.99		<0.01	
**Ethnicity** [d]												
Hispanic	1.00 (1.00, 1.01)	0.14	1.07 (0.98, 1.18)	0.09	1.00 (1.00, 1.01)	0.14	1.00 (0.99, 1.01)	1.00	1.04 (0.95, 1.15)	0.29	1.01 (0.98, 1.04)	0.58

	Any SDB		Types of SDB									
			CSA		OSA		OUSA		Multiple sleep apneas		Procedure-based cases	
	PR (Bonferroni-corrected 95% CI)	P-value	PR (Bonferroni-corrected 95% CI)	P-value	PR (Bonferroni-corrected 95% CI)	P-value	PR (Bonferroni-corrected 95% CI)	P-value	PR (Bonferroni-corrected 95% CI)	P-value	PR (Bonferroni-corrected 95% CI)	P-value
Non-Hispanic	**1.03 (1.02, 1.03)**	<0.01	1.02 (0.98, 1.05)	0.27	**1.02 (1.02, 1.03)**	<0.01	**1.03 (1.02, 1.03)**	<0.01	**1.03 (1.00, 1.07)**	0.03	**1.05 (1.04, 1.07)**	<0.01
P for interaction	<0.01		0.60		<0.01		0.20		0.99		<0.01	

Abbreviations: SDB=Sleep disordered breathing, CSA=Central Sleep Apnea, OSA=Obstructive Sleep Apnea, OUSA=Other/unspecified Sleep Apnea, AI/AN=American Indian/Alaskan Native, BAA= Black/African American, NHPI=Native Hawaiian/Pacific Islander, HVM=Health Vulnerability Module, PR=Prevalence ratios, CI=Confidence intervals.

Results from log-binomial models with reported outputs of exponentiated beta coefficients (prevalence ratios) and their Bonferroni-corrected 95% confidence intervals.

[a] Adjusted for sex, race, and ethnicity (Bonferroni-corrected p-critical value = 0.017).

[b] Adjusted for age category, race, and ethnicity (Bonferroni-corrected p-critical value = 0.017).

[c] Adjusted for age category, sex, and ethnicity (Bonferroni-corrected p-critical value = 0.008).

[d] Adjusted for age category, sex, and race (Bonferroni-corrected p-critical value = 0.025).

P for the interaction effect is the p-value of a likelihood ratio test comparing log-binomial models with and without an interaction term.

Bolded values indicate statistical significance (P<0.05).

NE- Not estimated.

medRxiv preprint doi: https://doi.org/10.1101/2025.10.01.25337104; this version posted October 2, 2025. The copyright holder for this preprint (which was not certified by peer review) is the author/funder, who has granted medRxiv a license to display the preprint in perpetuity.
This article is a US Government work. It is not subject to copyright under 17 USC 105 and is also made available for use under a CC0 license.

eTable 12. Sensitivity analysis for cross-sectional association of per 0.1-unit increase Environmental, Social, and Health Burden (ESHB), Environmental Burden Module (EBM), Social Vulnerability Module (SVM), and Health Vulnerability Module (HVM) ranks with sleep-disordered breathing (SDB), central sleep apnea (CSA), obstructive sleep apnea (OSA), and other/unspecified sleep apnea (OUSA), multiple sleep apneas, and procedure-based cases

	Model 1	Model 2	Model 3
	**PR (95% CI)**	**PR (95% CI)**	**PR (95% CI)**
**Any SDB**			
ESHB	1.00 (0.99, 1.00)	1.00 (1.00, 1.00)	**1.01 (1.01, 1.01)**
EBM	**0.95 (0.95, 0.95)**	**0.96 (0.96, 0.96)**	**0.98 (0.97, 0.98)**
SVM	**0.99 (0.99, 0.99)**	**0.99 (0.99, 0.99)**	**1.02 (1.01, 1.02)**
HVM	**1.05 (1.04, 1.05)**	**1.04 (1.03, 1.04)**	**1.02 (1.02, 1.02)**
**CSA**			
ESHB	0.99 (0.95, 1.02)	0.99 (0.95, 1.03)	1.02 (0.98, 1.06)
EBM	**0.92 (0.89, 0.95)**	**0.93 (0.90, 0.97)**	0.97 (0.93, 1.00)
SVM	0.97 (0.94, 1.01)	0.97 (0.94, 1.01)	1.02 (0.98, 1.06)
HVM	**1.07 (1.04, 1.11)**	**1.06 (1.03, 1.10)**	**1.05 (1.02, 1.09)**
**OSA**			
ESHB	0.99 (0.99, 1.00)	0.99 (0.99, 1.00)	1.01 (1.00, 1.01)
EBM	**0.94 (0.94, 0.95)**	**0.95 (0.95, 0.96)**	**0.97 (0.97, 0.97)**
SVM	**0.99 (0.99, 0.99)**	**0.99 (0.99, 0.99)**	**1.02 (1.01, 1.02)**
HVM	**1.04 (1.04, 1.05)**	**1.04 (1.03, 1.04)**	**1.02 (1.02, 1.02)**
**OUSA**			
ESHB	1.00 (1.00, 1.01)	**1.01 (1.00, 1.01)**	**1.01 (1.01, 1.02)**
EBM	**0.98 (0.97, 0.98)**	**0.98 (0.98, 0.99)**	0.99 (0.99, 1.00)
SVM	**0.98 (0.98, 0.99)**	**0.99 (0.98, 0.99)**	1.00 (1.00, 1.01)
HVM	**1.05 (1.04, 1.05)**	**1.04 (1.03, 1.04)**	**1.02 (1.02, 1.03)**
**Multiple sleep apneas**			
ESHB	**0.95 (0.92, 0.98)**	**0.95 (0.92, 0.98)**	0.99 (0.95, 1.02)
EBM	**0.83 (0.80, 0.86)**	**0.84 (0.81, 0.88)**	**0.89 (0.85, 0.92)**
SVM	0.99 (0.95, 1.02)	0.99 (0.95, 1.03)	**1.05 (1.01, 1.09)**
HVM	**1.05 (1.02, 1.08)**	**1.04 (1.00, 1.07)**	1.03 (1.00, 1.06)
**Procedure-based cases**			
ESHB	**1.03 (1.02, 1.04)**	**1.03 (1.02, 1.05)**	**1.05 (1.04, 1.06)**
EBM	**0.92 (0.91, 0.93)**	**0.93 (0.92, 0.94)**	**0.94 (0.93, 0.96)**
SVM	**1.05 (1.04, 1.07)**	**1.06 (1.04, 1.07)**	**1.09 (1.07, 1.10)**
HVM	**1.06 (1.04, 1.07)**	**1.05 (1.04, 1.06)**	**1.04 (1.03, 1.06)**

Abbreviations: SDB=Sleep disordered breathing, CSA=Central Sleep Apnea, OSA=Obstructive Sleep Apnea, OUSA=Other/unspecified Sleep Apnea,

ESHB=Environmental, Social, and Health Burden, EBM=Environmental Burden Module, SVM=Social Vulnerability Module, HVM=Health Vulnerability Module,

PR=Prevalence ratios; CI=Confidence intervals.

medRxiv preprint doi: https://doi.org/10.1101/2025.10.01.25337104; this version posted October 2, 2025. The copyright holder for this preprint (which was not certified by peer review) is the author/funder, who has granted medRxiv a license to display the preprint in perpetuity.
This article is a US Government work. It is not subject to copyright under 17 USC 105 and is also made available for use under a CC0 license.

Results from log-binomial models with reported outputs of exponentiated beta coefficients (prevalence ratios) and their 95% confidence intervals.

Model 1: Unadjusted; Model 2: Age category-adjusted; Model 3: Fully-adjusted for age category, sex, race, and ethnicity.

Bolded values indicate statistical significance (P<0.05).
